# Wide diversity in structure and expression profiles among members of the *Caenorhabditis elegans *globin protein family

**DOI:** 10.1186/1471-2164-8-356

**Published:** 2007-10-04

**Authors:** David Hoogewijs, Eva Geuens, Sylvia Dewilde, Andy Vierstraete, Luc Moens, Serge Vinogradov, Jacques R Vanfleteren

**Affiliations:** 1 Department of Biology and Center for Molecular Phylogeny and Evolution, Ghent University, B-9000 Ghent, Belgium; 2 Department of Biomedical Sciences, University of Antwerp, B-2610 Antwerp, Belgium; 3 Department of Biochemistry and Molecular Biology, Wayne State University School of Medicine, Detroit, Michigan 48201, USA

## Abstract

**Background:**

The emergence of high throughput genome sequencing facilities and powerful high performance bioinformatic tools has highlighted hitherto unexpected wide occurrence of globins in the three kingdoms of life. *In silico *analysis of the genome of *C. elegans *identified 33 putative globin genes. It remains a mystery why this tiny animal might need so many globins. As an inroad to understanding this complexity we initiated a structural and functional analysis of the globin family in *C. elegans*.

**Results:**

All 33 *C. elegans *putative globin genes are transcribed. The translated sequences have the essential signatures of single domain *bona fide *globins, or they contain a distinct globin domain that is part of a larger protein. All globin domains can be aligned so as to fit the globin fold, but internal interhelical and N- and C-terminal extensions and a variety of amino acid substitutions generate much structural diversity among the globins of *C. elegans*. Likewise, the encoding genes lack a conserved pattern of intron insertion positioning. We analyze the expression profiles of the globins during the progression of the life cycle, and we find that distinct subsets of globins are induced, or repressed, in wild-type dauers and in *daf-2(e1370)*/insulin-receptor mutant adults, although these animals share several physiological features including resistance to elevated temperature, oxidative stress and hypoxic death. Several globin genes are upregulated following oxygen deprivation and we find that HIF-1 and DAF-2 each are required for this response. Our data indicate that the DAF-2 regulated transcription factor DAF-16/FOXO positively modulates *hif-1 *transcription under anoxia but opposes expression of the HIF-1 responsive globin genes itself. In contrast, the canonical globin of *C. elegans*, ZK637.13, is not responsive to anoxia. Reduced DAF-2 signaling leads to enhanced transcription of this globin and DAF-16 is required for this effect.

**Conclusion:**

We found that all 33 putative globins are expressed, albeit at low or very low levels, perhaps indicating cell-specific expression. They show wide diversity in gene structure and amino acid sequence, suggesting a long evolutionary history. Ten globins are responsive to oxygen deprivation in an interacting HIF-1 and DAF-16 dependent manner. Globin ZK637.13 is not responsive to oxygen deprivation and regulated by the Ins/IGF pathway only suggesting that this globin may contribute to the life maintenance program.

## Background

Globins constitute a large superfamily of heme-binding proteins that are encountered in all the kingdoms of life [1,2]. Globin polypeptides typically comprise 145 to 155 amino acid residues that are folded into a characteristic three dimensional structure, the globin fold: six to eight α-helical segments connected by short loops form a helical sandwich that encloses non-covalently bound heme within a cavity of hydrophobic residues. Single globin units can aggregate or fuse with each other or with other polypeptide chains to form a bewildering complexity of quaternary structures including monomers, dimeric, tetrameric and polymeric forms, multi-subunit and multi-domain, multi-subunit proteins, ranging from 17 to 3600 kDa in size [3]. The evolution of these high molecular weight structures is likely linked with their extracellular occurrence to avoid elimination from the extracellular fluid by excretory processes. The A and G helices of many annelid and vestimentiferan globins contain cysteine residues that readily form cystine bridges with other globin units, and linker polypeptides forming high molecular weight aggregates [4-7]. Concatenation of globin domains resulting from gene duplication also results in increases in Mr and can be combined with further aggregation. These structures are found in nematodes, some bivalve molluscs and crustaceans [8-10] (for a comprehensive review see [3]).

Globins have been called respiratory proteins because transport and facilitation of diffusion of oxygen to the mitochondria are the predominant and first established functions of vertebrate globins. A role in NO metabolism was only recently demonstrated for these proteins [11-14]. Functions identified for nonvertebrate globins are much more diverse and also include oxygen sensing, storage or scavenging reactions with sulphate, and oxidase and peroxidase activities (for an extensive review see [3]).

Nematodes express several globins, including cellular, perienteric and cuticular isoforms. Some of them likely function in facilitating respiration. *Enoplus brevis *expresses large quantities of globin in the pharynx which enables this species to feed efficiently at low *P*o_2_. *Nippostrongylus brasiliensis*, an intestinal parasite of rat and mouse contains 2 globin isoforms, one localized in the body wall and the other in the cuticle. Both globins have oxygen affinities 100-fold higher than the rodent host's hemoglobins and likely serve to shuttle oxygen from the worm's gut to its tissues [15]. Other nematode globins have unprecedented functions. The globin molecule which is abundantly present in the perienteric fluid of *Ascaris lumbricoides *is an octamer of didomain globin polypeptides. It binds molecular oxygen more than 10,000 fold stronger than vertebrate myoglobin [16], excluding a function in O_2 _transport. Its function is still unknown. Suggested roles include providing heme to the oocytes (nematodes are unable to synthesize heme and must acquire it from their food ([17,18]), assisting sterol biosynthesis [19], acting as a NADPH-dependent reductase for cytochrome c [19] and scavenging oxygen by oxygenating NO [20]. *Mermis nigrescens*, a parasite nematode of grass hoppers, has two cellular globin isoforms, that are 84% identical. The body isoform presumably acts as a myoglobin, but the other isoform is very highly expressed in the ocellus of mature phototactic females, where it forms intracellular crystals and likely acts as a shading pigment [21].

The finding of a first globin gene in the genome of *C. elegans *more than a decade ago [22,23] was surprising since these small animals were generally thought to rely entirely on diffusion for gaseous exchange. However, careful *in silico *analysis of the genome of *C*. *elegans *has revealed the presence of more than 30 proteins that are predicted to exhibit or to contain globin or globin-like domains and could be aligned so as to fit determinants of the globin fold [24]. *C. elegans *is a small (1.2–1.5 mm long and 50–70 μm wide) free-living soil nematode. This species has two sexes, hermaphrodites and males, but the latter comprise only about 0.1%–0.2% of the population. The life cycle consists of four larval stages (referred to as L1-L4) and the adult stage, separated by molts. When exposed to unfavorable conditions of crowding, high temperature and scarcity of food, a second stage larva can enter diapause and molt to a dauer larva, a facultative and specialized L3 stage that does not feed, is resistant to harsh environmental conditions and can survive up to eight times the normal 3-week life span of animals that have bypassed this stage. Entry and exit from the dauer stage is controlled by interacting neuroendocrine signaling, including TGF-β, Ins/IGF-1, cyclic nucleotide and gonadal signaling. The metabolic adaptations of dauer diapause are controlled by Ins/IGF-1 signaling. Insulin-like peptides can bind on the unique Ins/IGF-1-like receptor encoded by the *daf-2 *gene. Upon binding insulin ligand, DAF-2 (genes are written in italics; their protein products in non-italicized capitals) activates a signaling cascade that phosphorylates the FOXO transcription factor DAF-16. DAF-16 resides in the nucleus but relocates to the cytoplasm and is inactive when phosphorylated. Thus DAF-16 is negatively regulated by DAF-2. In the nucleus DAF-16 can activate an enhanced life maintenance program, characterized by alterations in energy and intermediate metabolism, elevated stress resistance and prolonged survival. Animals carrying partial loss-of-function alleles (e.g. *e1370*) of *daf-2 *form dauers constitutively. *e1370 *is a temperature-sensitive allele permitting normal development at the permissive temperature (15°C). When the culture temperature is raised to 25°C after development to L3, the animals grow to adults that exhibit several characteristics of the dauer stage, and live twice as long as wild-type animals [25-27]. A signal from the gonad also regulates life span in *C. elegans*. A lipophilic hormone synthesized in the somatic gonad by the cytochrome P450 homolog DAF-9 binds and activates the nuclear receptor DAF-12 in responsive cells, where DAF-12 and DAF-16 co-regulate transcription of downstream effectors of diapause. This pathway also enables integration of signals from the reproductive system with DAF-16 activation to modulate life span [28-30].

Here, we investigate the structure of the globin genes and the amino acid sequence of the gene products by computational analysis of the genomes of *C. elegans *and of *C. briggsae *[31], one of its closest relatives, and by sequence analysis of the transcribed messages. We examine the expression profile of the globin family during the progression of the life cycle and we find that distinct subsets of the globins are differentially regulated in dauers and long-lived *daf-2(e1370) *mutant animals. Several globin genes are upregulated following anoxia and we find that both HIF-1 and DAF-2 function is required for this response to oxygen deprivation.

## Results

### Identification of putative globins and validation of the covalent structures

**Figure 1:**
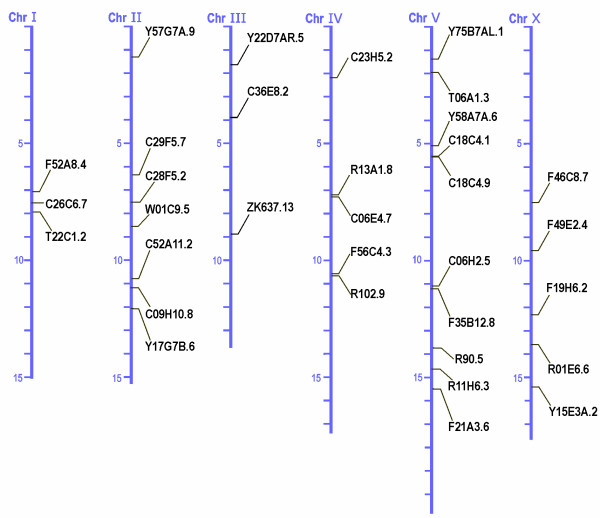
Chromosomal distribution of the *C. elegans *globin genes.

A previous report by Hoogewijs et al. [24] revealed the presence of 35 putative globins in the genome of *C. elegans*. A more stringent analysis using the sequence-structure homology recognition tool FUGUE [32] reduced this number to 33, still a number by far not reached in any other organism studied to date [1], second only to the larvae of the insect *Chironomus *with over 40 globin genes [33]. All putative globin genes have orthologous genes in *C. briggsae *with identities ranging from 67.8 % to 99.7 %. Screening of the recently published draft genomic sequence of *C. remanei *also indicated the presence of an ortholog for each candidate globin gene. The putative globin genes are distributed over all 6 chromosomes and no clusters are found, with the exception of C18C4.1 and C18C4.9 which are separated by only 4 kb. Ten of the 33 putative globins are located on chromosome V (Fig. 1).

Comparison of the predicted *C. elegans *and *C. briggsae *orthologs was very helpful in delineating potentially wrongly predicted portions. The globin orthologs from these species have BLAST E-values ranging from about e-69 to e-215 and the aligned sequences show very few sequence changes. Portions that are conspicuously dissimilar delineate inaccurate sequence prediction. We found that all the genes listed in Table 1 are transcribed, but that 7 genes are partially wrongly predicted. Internal annotation errors could be readily corrected by RT-PCR. 5' and 3' RACE experiments were needed for correcting N- and C-terminal annotation errors. The predicted ORFs for the genes Y58A7A.6 and F21A3.6 were corrected in successive WormPep versions in WormBase in the course of this study as well. The ORF for the gene C18C4.1 was also changed, but we found that it was still mispredicted. A comparison of the C26C6.7 predicted genomic sequence and our cDNA sequence revealed that the first Met residue in the N-terminal sequence of C26C6.7 predicted by Genefinder is incorrect. The predicted sequence of C28F5.2 was corrected at the amino terminus (extra first exon and different position of the second exon). Several of the wrongly predicted genes needed multiple corrections. C18C4.1 needed correction at the N-terminus and insertion of an additional internal exon, and a large C-terminal portion of the annotated gene turns out to be part of a 3' UTR, due to a frame shift caused by a 10 bp insertion compared to the predicted sequence. The predicted sequence of T06A1.3 needed an internal correction in the F helix and incorporation of the annotated T06A1.4 gene at its amino terminus. The predicted sequence of Y22D7AR.5 (lacking an F, G and H helix) was corrected at its C-terminus by subsuming Y22D7AR.4 into it. The *C. briggsae *ortholog found in WormBase also lacks F, G and H helix, but its TWINSCAN prediction (cb25.fpc2587.0.059.a) aligns very well with our corrected sequence. Altogether, the predictions for the genes Y58A7A.6, F21A3.6, C18C4.1, C26C6.7, C28F5.2, T06A1.3 and Y22D7AR.5 were fully corrected. Eventually we could validate the primary structures of all 33 putative globins. Interestingly, several of these show homology to vertebrate neuroglobin and cytoglobin, as revealed by BLAST searches (Table 1).

**Table 1:** Overview of structural characteristics

**Gene**	**Accession no.**	***C. briggsae *homolog**	**Motif/similarity**	**Total length**	**Pre A helix**	**Post H helix**	**GH Inter-helix**
*C06E4.7*	AAA82476	*CBG05824*	globin-like	230	26	26	21
*C06H2.5*	CAA99771	*CBG23115*	globin	209	32	25	
*C09H10.8*	CAA90439	*CBG02965*	globin-like	344	-	120	22
*C18C4.1*	EF471982	*CBG09371*	globin	358	34	162	
*C18C4.9*	AAK52180	*CBG09369*	globin	389	195	40	
*C23H5.2*	AAP82661	*CBG10551*	ngb (Xenopus) 5.4 e-07	278	5	115	
*C26C6.7*	EF471981	*CBG11881*	cygb (human) 3 e-05	404	193	22	
*C28F5.2*	EF471980	*CBG13047*	globin	194	10	42	
*C29F5.7*	AAC46826	*CBG02622*	globin	198	26	16	
*C36E8.2*	CAA84649	*CBG03635*	globin-like	254	41	71	
*C52A11.2*	CAA86764	*CBG03023*	globin	266	30	56	21
*F19H6.2*	CAA92164	*CBG00138*	ngb (human) 2.8 e-09	231	48	31	
*F21A3.6*	CAB04152	*CBG18593*	cygb (human) 8.2 e-12	236	83	-	
*F35B12.8*	CAA98468	*CBG23302*	globin-like	234	34	48	
*F46C8.7*	AAF99945	*CBG16720*	globin-like	322	59	99	
*F49E2.4*	CAA86423	*CBG16082*	cygb (Danio) 7.9 e-06	216	12	16	40
*F52A8.4*	CAA95823	*CBG11915*	cygb (human) 0.00014	238	68	11	
*F56C4.3*	CAE17840	*CBG04428*	ngb (Xenopus) 4.1e-05	214	-	47	
*R01E6.6*	CAA92187	*CBG07422*	globin-like	311	94	75	
*R102.9*	CAE17922	*CBG17640*	globin	196	15	-	32
*R11H6.3*	CAB07647	*CBG04577*	cygb (human) 0.06	387	132	87	
*R13A1.8*	AAA81477	*CBG05809*	globin	342	159	21	
*R90.5*	CAD31696	*CBG09511*	ngb (Xenopus) 0.006	322	141	24	
*T06A1.3*	EF471978	*CBG24799*	globin-like	189	31	-	
*T22C1.2*	CAA99921	*CBG08252*	globin	183	25	6	
*W01C9.5*	CAA90269	*CBG00571*	globin	224	22	51	
*Y15E3A.2*	CAB60330	*CBG07681*	globin-like	217	55	23	
*Y17G7B.6*	CAA19459	*CBG21021*	cygb (human) 0.01	216	36	20	
*Y22D7AR.5*	EF471979	*CBG11736*	ngb (human) 0.0001	272	6	103	17
*Y57G7A.9*	AAC26293	*CBG07112*	globin-like	169	8	6	
*Y58A7A.6*	AAK84620	*CBG08670*	globin	230	88		
*Y75B7AL.1*	AAK68603	*CBG06424*	globin	542	372	8	
*ZK637.13*	CAA77458	*CBG06867*	globin	159	-	5	

### Wide diversity in the primary structures

**Figure 2:**
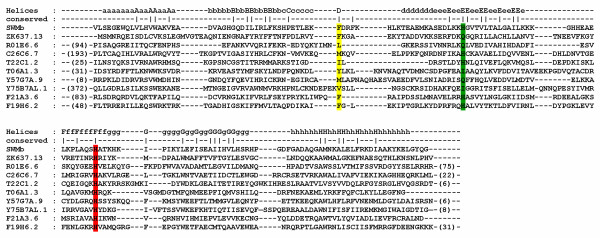
Alignment of 9 representative globins and *Sperm whale *(SW) myoglobin. ZK637.13 is the canonical globin. It was the first reported globin species in *C. elegans *and features all characteristics of a standard globin. The next 6 globins have an unusual amino acid residue at CD1. Y75B7AL.1 is a chimeric protein consisting of a G-coupled receptor domain (not shown) and a globin domain. F21A3.6 and F19H6.2 show homology with mammalian cytoglobin and neuroglobin, respectively. Numbers between brackets indicate the number of amino acids preceding and following the globin domain. The residues at position CD1, E7 and F8 are marked in yellow, green and red, respectively.

The profiles of all candidate globins are listed in Table 1. The total length ranges from 159 (ZK637.13) to 542 (Y75B7AL.1) amino acid residues. A typical globin domain comprises ~150 residues. The increase in length is caused by N- and/or C-terminal and, more exceptionally, internal extensions. A sequence alignment of all 33 globins is provided in additional file 1. Nine sequences were extracted from this alignment block and are shown in Fig. 2 to illustrate structural features discussed in the text. All sequences can be aligned to fit the globin fold, displaying F8His and several other globin signatures, including CD1Phe, E7His or Gln. However, six putative globins have Leu (R01E6.6), Ile (T22C1.2), Tyr (T06A1.3), Met (C26C6.7, Y57G7A.9) or Val (Y75B7AL.1) instead of Phe at CD1 and four have Ile (T06A1.3, Y75B7AL.1, C09H10.8) or Val (C06E4.7) instead of His or Gln at E7. C-terminal extensions (up to 120 amino acids in C09H10.8) are found in 10 putative globins, whereas N-terminal extensions (372 amino acids inY75B7AL.1) are found in 12 putative globins. Interestingly, several prediction programs (TMHMM server v. 2.0 [34], TMpred [35] and DAS [36]) revealed that the N-terminal portion of Y75B7AL.1 has the structural characteristics of a G-coupled receptor-like containing 7 putative transmembrane helices. Internal GH interhelical extensions of unusual length are seen in F49E2.4 (40aa), R102.9 (32aa), C52A11.2 (21aa), C06E4.7 (21aa), C09H10.8 (22aa) and Y22D7AR.5 (17aa). T19C4.5 displays an alternative splice variant and features F8Asn instead of F8His, whereas its *C. briggsae *homolog contains F8His. Moreover amino acid sequence comparison of both orthologs reveals that the globin domain differs only in 6 aa and FUGUE still defines it as a globin. As the functionally essential histidine at helix position F8 has been replaced by asparagine, precluding proper heme binding, T19C4.5 was omitted from our globin set.

### No conservation of intron insertion patterns

**Figure 3:**
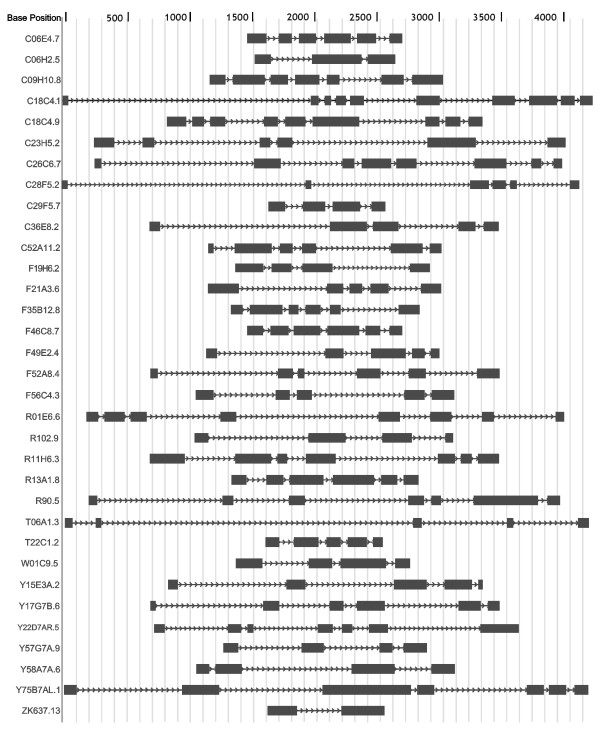
Genomic structures.

**Figure 4:**
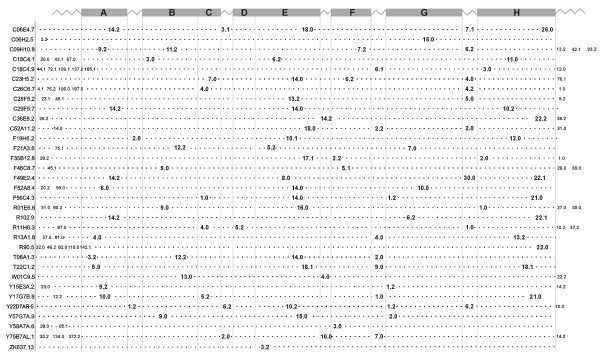
Lack of conservation in the intron insertion positions. Phase 0 introns (55%) are inserted between 2 successive codons; phase 1 (9%) and phase 2 introns (36%) are inserted following the first and second base of a codon, respectively.

Vertebrate globins genes typically have two introns inserted at conserved positions B12.2 (intron located between codon positions 2 and 3 of the 12^th ^amino acid of globin helix B) and G7.0, whereas plant globin genes have an extra intron at E15.0 and most insect globin genes contain no intervening sequences [37,38]. We have compared the exon/intron patterns among all 33 putative globin genes in Fig. 3 and Fig. 4. Strikingly, no conservation is discernible neither in the number of introns (1–9) and exons (2–10) nor in the exon/intron boundaries. Examination of the intron insertion positions showed an even more remarkable diversity. The number of introns that interrupt the globin domain ranges from 1 (ZK637.13) to 5 (C06E4.7); 1–5 and 1–3 introns are inserted in the pre-A and post-H portions of the proteins, respectively. In the globin domain, 76 different insertion positions can be found of which most are unprecedented. Many positions are found in interhelical segments, 9 in the GH interhelix from which 5 are located in GH interhelical extensions. Only one globin gene (F21A3.6) features both conserved vertebrate insertion sites B12.2 and G7.0. Fourteen of the 33 putative globin genes display 3 intron insertions in the globin domain, 7 of them feature 2 interruptions in the globin domain.

### Developmental expression pattern

**Figure 5:**
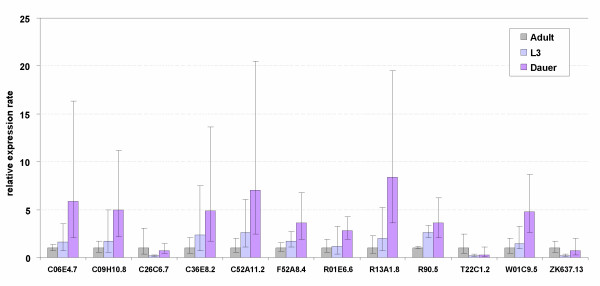
Levels of globin expression in third stage larvae (L3) and the alternative dauer (diapause) stage, relative to expression in young adult animals. The expression ratio's are the average values from 3 replicate cultures (biological repeats). Bars indicate the 95% confidence interval of the mean. Only globins that showed a significant (borderline for C36E8.2) difference in one of the three comparisons are displayed.

To establish the pattern of globin gene expression throughout the life cycle of the animal, we studied highly synchronized developmental staged *C. elegans *using RT-PCR, including embryo's (eggs), L1, L2, dauers, L3, L4, and young adults (1–2 days post the L4 to adult molt. We found that a large portion of globins are expressed through all stages of development (Additional file 2). Differences in expression levels between developmental stages were evaluated more accurately using quantitative RT-PCR. We examined the expression levels of the globin family in L3 and dauers, relative to wild-type young adults. Biological replicates were done on 3 independent worm cultures. Several globin genes (C06E4.7, C09H10.8, C36E8.2, C52A11.2, F52A8.4, R01E6.6, R13A1.8, R90.5, and W01C9.5) are similarly upregulated in L3 and dauers relative to young adults, although some reach significance in dauers only (Fig. 5). Many genes exhibited more than 2- fold upregulation but didn't reach statistical significance because strong upregulation was only seen in 2 biological replicates (Additional file 3). Remarkably, we observed a significant downregulation in L3 stage relative to young adults for C26C6.7, T22C1.2 and ZK637.13. A similar trend was seen in dauers. Interestingly, C26C6.7 was the only globin which was expressed at a significantly higher level in dauers relative to L3.

We also used quantitative real-time RT-PCR experiments to compare the relative abundance of all 33 globins in wild type adults (Table 2). Results demonstrate that T22C1.2 and ZK637.13 are expressed at substantially higher levels. The difference with the other globins ranges within 1–3 orders of magnitude.

**Table 2:** Relative expression levels. Values are average percentages and standard deviations from 6 independent experiments

**Globin gene**	**Relative expression**	**Standard Deviation**
C06E4.7	0.267	0.174
C06H2.5	0.262	0.030
C09H10.8	0.272	0.094
C18C4.1	0.328	0.446
C18C4.9	2.245	1.626
C23H5.2	0.304	0.147
C26C6.7	0.196	0.171
C28F5.2	0.554	0.490
C29F5.7	1.799	0.805
C36E8.2	1.611	1.208
C52A11.2	0.279	0.300
F19H6.2	1.235	0.641
F21A3.6	0.421	0.170
F35B12.8	0.431	0.380
F46C8.7	0.164	0.044
F49E2.4	0.604	0.196
F52A8.4	0.339	0.096
F56C4.3	1.103	0.298
R01E6.6	2.697	0.311
R102.9	0.052	0.043
R11H6.3	0.463	0.180
R13A1.8	0.449	0.149
R90.5	0.558	0.349
T06A1.3	0.395	0.309
T22C1.2	43.659	8.788
W01C9.5	0.547	0.269
Y15E3A.2	0.237	0.166
Y17G7B.6	1.443	0.508
Y22D7AR.5	0.191	0.223
Y57G7A.9	0.172	0.083
Y58A7A.6	0.981	0.468
Y75B7Al.1	4.329	0.852
ZK637.13	31.409	10.324

### Anoxia-induced globin expression

**Figure 6:**
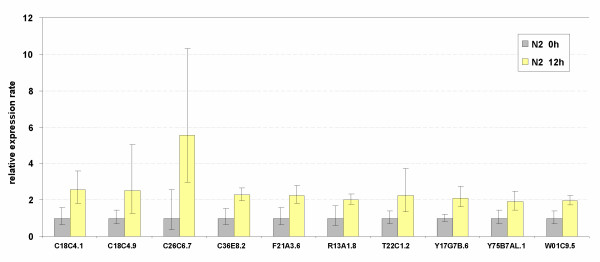
Ten genes are upregulated in young adult wild-type animals following 12 h of anoxia. The expression ratio's are the average values from 3 replicate cultures (biological repeats). Bars indicate the 95% confidence interval of the mean.

To investigate which globins might be upregulated in response to oxygen deprivation we subjected young adult worms (1–2 days of adult age) to anoxic conditions for 12 h. RNA was subsequently prepared and amplified by quantitative RT-PCR. A separate viability assay showed that more than 90% of the worm population remained viable under these conditions, and they recovered within 24 h under normoxic conditions. Our criteria for differential regulation were: (1) two-fold difference in expression with matched samples, and (2) non-overlapping 95% confidence intervals. Six genes (C26C6.7, F21A3.6, Y17G7B.6, R13A1.8, C18C4.1 and C36E8.2) met both these criteria and are referred to as anoxia-responsive. T22C1.2 and C18C4.9 exhibited greater than 2-fold upregulation by anoxia but didn't reach statistical significance (p < 0.06 and p < 0.07, respectively) as 1 biological replicate showed only moderate upregulation. Expression of W01C9.5 and Y75B7AL.1 was induced 1.96 and 1.89, respectively. These four genes are referred to as likely anoxia-responsive (Fig. 6). None of the 33 globin transcripts in N2 worms showed reduction in expression under anoxia.

Three (C26C6.7, F21A3.6 and Y17G7B.6) out of the 6 anoxia-responsive genes displayed sequence similarity to vertebrate cytoglobin, whereas none of the five genes that showed homology to vertebrate neuroglobin was induced by oxygen deprivation (Additional file 4). Interestingly, a similar lack of hypoxic response was reported for vertebrate neuroglobin [39-41].

### HIF-1 dependent globin genes

**Figure 7:**
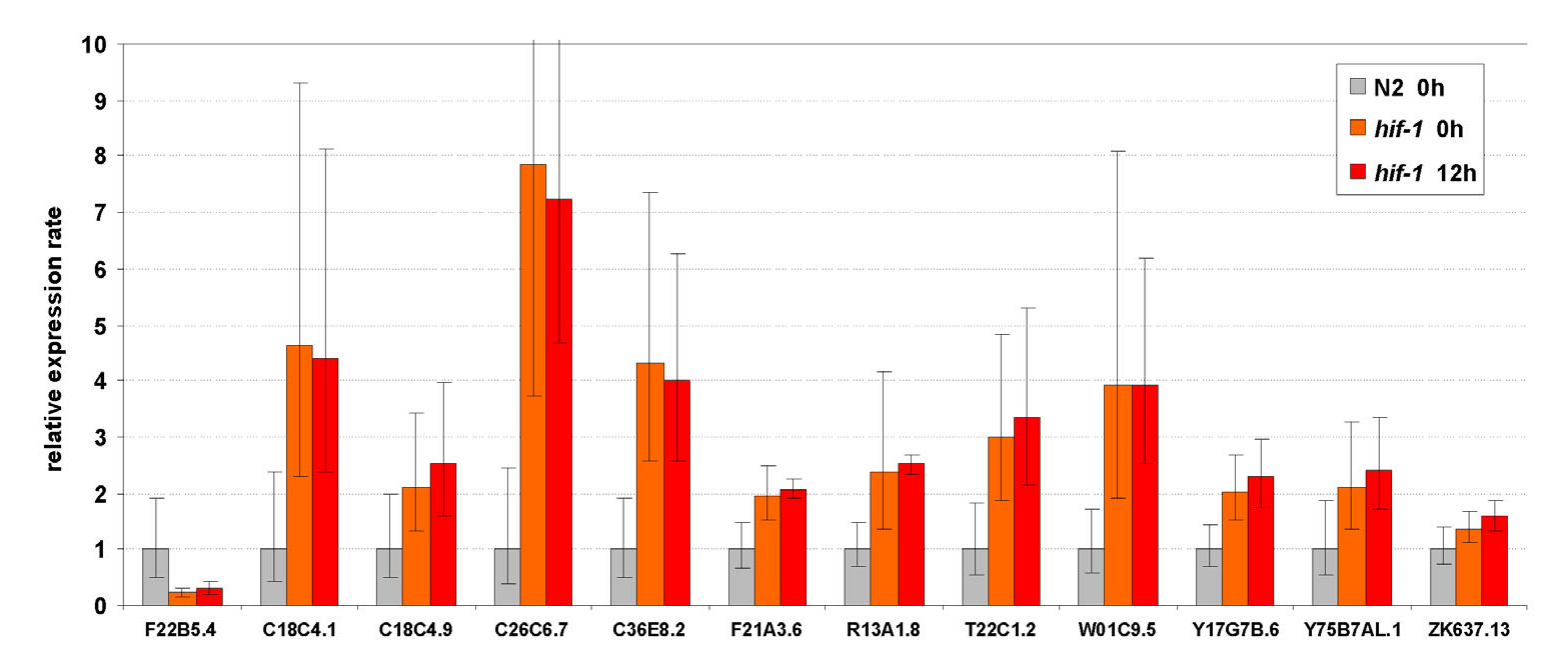
*hif-1 *mediates induction of the anoxia responsive genes. The expression ratio's are the average values from 3 replicate cultures (biological repeats). Bars indicate the 95% confidence interval of the mean. F22B5.4 is an established hypoxia responsive gene of unknown function [43] and is included as a positive control. Globin ZK637.13 is not sensitive to anoxia and was included as a negative control.

To find out whether HIF-1 (hypoxia-inducible factor) was required for regulation of the anoxia-responsive genes, we measured the expression levels of the proven and likely anoxia-responsive genes listed in Fig. 6 in *hif-1 *mutants grown in normoxic and anoxic conditions. ZK637.13, which is anoxia-insensitive in wild-type worms but strongly induced in adult *daf-2(e1370)*, was also included in this assay. The non-globin, HIF-1 dependent gene F22B5.4 (unknown function) was used as a positive control [42,43]. F22B5.4 was expressed at lower levels under both normoxia and hypoxia in *hif-1 *defective animals, as expected [43], but to our surprise all hypoxia-sensitive globins tended (statistically significant for C26C6.7, T22C1.2, F21A3.6, C36E8.2, W01C9.5 and Y17G7B.6) to be expressed at higher levels in *hif-1 *compared to wild-type animals. However none of them was differentially regulated under anoxia in *hif-1 *defective mutant worms, indicating that they are HIF-1 dependent (Fig. 7).

Additionally, candidate regulatory regions were examined for putative hypoxia-responsive sequence elements (HREs). Results indicate the presence of candidate HIF-1 binding elements in the genomic region of all anoxia-induced globin genes.

### DAF-2- and DAF-16-regulated globin gene expression

**Figure 8:**
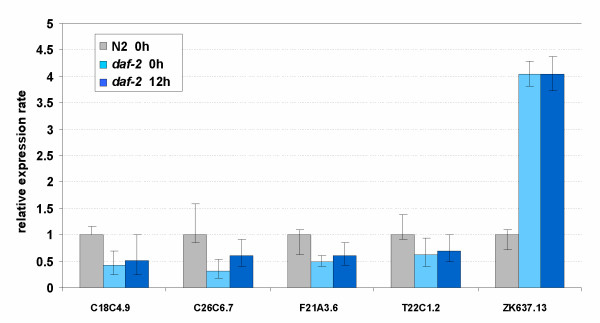
Expression levels of globin genes in *daf-2 *young adults under normoxia or following 12 h exposure to anoxic conditions, relative to wild-type young adult worms under normoxia. The expression ratio's are the average values from 3 replicate cultures (biological repeats). Bars indicate the 95% confidence interval of the mean. Only globins that showed a significant (borderline for T22C1.2) difference normoxia N2 vs. normoxia *daf-2 *are displayed.

Since *daf-2(e1370) *mutant worms are hypoxia tolerant [44,45], we anticipated that some globin genes might be constitutively upregulated in these animals. To our surprise we found only minor changes in transcription levels compared to wild-type worms (Additional file 5), except for ZK637.13 which was significantly upregulated by 4-fold. None of the HIF-1 dependent globin genes was significantly induced by anoxia in the *daf-2 *animals. Three globin genes (F21A3.6, C18C4.9 and C26C6.7) were significantly downregulated under normoxic conditions (Fig. 8).

**Figure 9:**
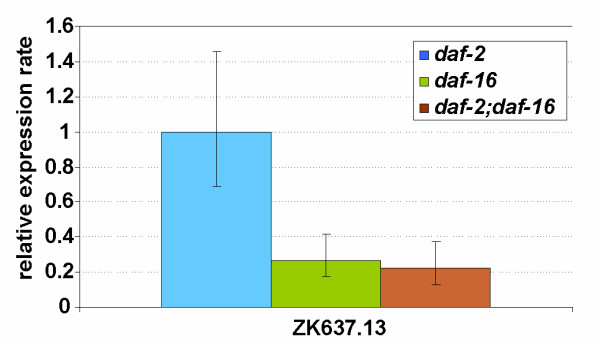
Expression levels of globin genes in *daf-16 and daf-2;daf-16 *young adults relative to *daf-2 *young adult worms. The expression ratio's are the average values from 3 replicate cultures (biological repeats). Bars indicate the 95% confidence interval of the mean.

To understand how DAF-2 exerts its effect on ZK637.13 we compared the expression level of this globin in *daf-16 *and *daf-2;daf-16 *mutant worms relative to *daf-2 *(Fig. 9). We found that the expression of ZK637.13 was reduced by 4-fold in *daf-16 *and *daf-2;daf-16 *animals indicating that ZK637.13 is regulated by DAF-2 in a DAF-16 dependent fashion. Computational analysis of the ZK637.13 genomic region detected the presence of a DAF-16 binding element at position -350, providing additional support for DAF-16-mediated regulation.

**Figure 10:**
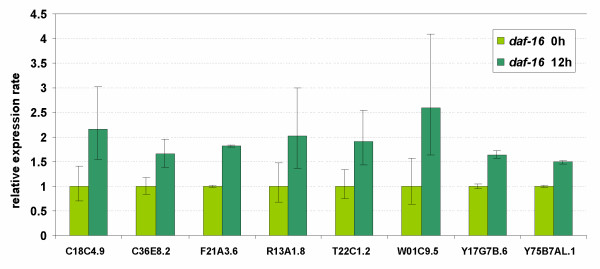
Expression levels of globin genes in *daf-16 *young adults following 12 h exposure to anoxic conditions relative to normoxic conditions. The expression ratio's are the average values from 3 replicate cultures (biological repeats). Bars indicate the 95% confidence interval of the mean. Only globins that showed a significant (borderline for R13A1.8) difference are displayed.

Since the HIF-1 dependent globin genes were not induced by anoxia in *daf-2 *mutants we expected that DAF-16 would not mediate or rather oppose their induction upon anoxia. We found that all anoxia inducible globin genes are upregulated in *daf-16 *animals under anoxic conditions, albeit at a lower level relative to wild-type worms (statistically significant and more than 2-fold for C18C4.9, W01C9.5; significant and less than 2-fold for C36E8.2, T22C1.2, F21A3.6 and Y17G7B.6; borderline significant for R13A1.8, Fig. 10).

### DAF-16 modulates *hif-1 *expression

**Figure 11:**
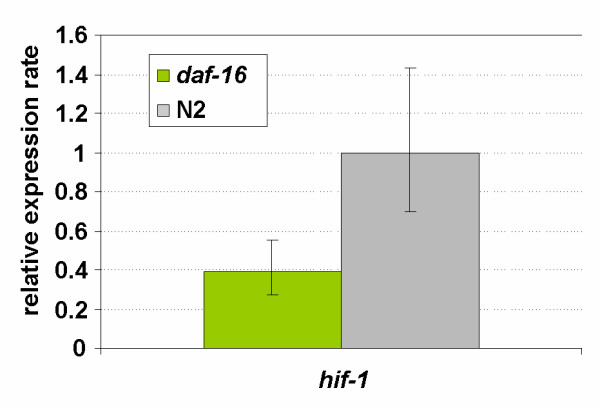
Expression level of *hif-1 *under anoxic conditions in *daf-16 *young adults relative to N2 young adult worms. The expression ratio's are the average values from 4 replicate cultures (biological repeats). Bars indicate the 95% confidence interval of the mean.

To learn more about the role of DAF-16 in the response to anoxia we measured the expression of *hif-1 *in *daf-16(m26) *and wild-type animals. *daf-16(m26) *is a very severe mutation that nearly fully disrupts the function of DAF-16. We found that transcription of *hif-1 *in the animals lacking *daf-16 *activity was lower under normoxia in each of four independent trials, but this difference was not statistically significant (not shown). However, expression of *hif-1 *was reduced by more than 2.5-fold (P < 0.025) under anoxic conditions in these animals (Fig. 11).

## Discussion

### Structural diversity

The finding of a globin sequence in the genome of *Caenorhabditis elegans *was unexpected because it was generally felt that due to the small size of this organism sufficient quantities of oxygen could reach the sites of oxygen consumption by simple diffusion, and for about a decade it was thought that globin ZK637.13 was the only globin species expressed in *C. elegans *[22,23]. However, the completion of the genome sequence of this species and the development of powerful gene prediction tools has now led to the identification of at least 33 putative globin genes that are all expressed. So, the paramount question arises whether these globins have distinct functional properties, or represent structural variations of the core globin and exhibit large redundancy. Usually such multigene families cluster together and the individual members become substrates for purifying or positive selection depending on the evolving function. Chironomid larvae live in low oxygen habitats and selection likely favored high copy number of similar genes to synthesize more hemoglobin. Several of these are stage-specific [33,46]. Because of the small size and terrestrial habitat of *C. elegans *it is likely that most globins in this species were selected for reasons other than enhancing hemoglobin synthesis capacity. The low transcript abundance of the globin genes also argues against such a role. Even globin ZK637.13, which has the second largest transcript abundancy is expressed in a subset of cells [47]. We found no evidence of recent gene duplication events, and the huge diversity seen in the primary structures of the proteins suggests a long evolutionary history. Taken together, these aspects of low expression and huge sequence diversity may indicate that most, if not all, of these globin genes are cell or stage specific, and that they have diverged to perform specialized functions.

The wide diversity in the gene structures, as shown by the marked variability of intron insertion patterns is in line with this view. Vertebrate globins genes typically have two introns inserted at conserved positions B12.2 and G7.0, whereas plant globin genes have an extra intron at E15.0. It was speculated that the 4 exons, 3 introns arrangement is ancestral and that evolution sometimes led to elimination of introns, particularly in bacteria, yeast and some insects [48,49]. As the positions of these canonical introns appear to divide the globin gene into functional domains, it was hypothesized that they witness the joining of functional exons to create the globin core unit early in evolution [48,50]. Although later studies demonstrated that there is no such general correlation of exon boundaries in genes with domain boundaries in proteins [51], it was thought that introns in eukaryotic genes witness the gene organization of the last universal common ancestor of cellular life, and that evolution sometimes led to their total (prokaryotes) or partial (eukaryotes) elimination [48,52]. This introns-early view was challenged by the intron-late hypothesis [53] which holds that introns in eukaryote genomes evolved after the split of prokaryotes and eukaryotes. This debate still persists [54,55]. The central question is how the complex eukaryotic spliceosomal machinery could have evolved. Self-splicing group I and group II introns are found in prokaryotes, but spliceosomal introns are a hallmark of eukaryotic genomes. A reconciling hypothesis holds that spliceosomal introns co-evolved with the origin of the eukaryote ancestor: an archaebacterial host acquired an α-proteobacterial symbiont (the mitochondrial ancestor). Group II introns from this symbiont invaded the hosts' genome and evolved to spliceosomal introns [56,57]. This initial intron invasion would have ended when the original intron-encoded protein was no longer required and was exposed to mutational demise. It is likely that introns that are inserted at very conservative positions throughout the evolution (e.g. B12.2, G7.0 and E15.0 in globin genes) are descendants of these founder introns. These, and many other introns have been lost and many other introns have been acquired more recently in nematodes. It is thought that these introns were gained by a fundamentally different process, likely reverse splicing of preexisting introns [54,58,59]. The lack of any conspicuous pattern of introns positioning in the globin genes of *C. elegans *reflects a dynamic pattern of intron insertion events, consistent with this hypothesis. Minor variability in intron positioning has been described for the cytochrome P450 [60] and DEAD helicase gene families [61] of *C. elegans*, however. The mechanisms controlling the stringent or more relaxed exon-intron pattern is currently not understood. A comprehensive analysis of the evolutionary history of the globin protein family in the genus *Caenorhabditis *will be described elsewhere.

### Anoxia-responsive globins

As a first approach to establish their functions, we have studied the expression profiles of these putative globins under normoxia and anoxia. *C. elegans *is a soil inhabiting nematode which relies on oxygen consumption by the mitochondria to maintain normal metabolic function. In the laboratory the worms are usually grown on agar plates, where they are exposed to normal atmospheric air, which contains 21 % oxygen or approx. 300 mg oxygen/L air. Oxygen supply will be lower in moist soil, mainly due to the poor solubility of oxygen in water, which is reduced to approx. 9.1 mg/L water at 20°C. *C. elegans *is able to maintain a constant metabolic rate when the oxygen concentration in air is lowered to approx. 3.6 %, and reduces respiration by 50% at 1 % oxygen (or 51.4 and 14.3 mg oxygen per liter air, respectively [62]). Thus water-logged or flooded soils rapidly become hypoxic and, not surprisingly, *C. elegans *has developed a response to cope with reduced oxygen supply. The animals can continue to develop and reproduce down to 0.1–0.25 % gaseous oxygen by activation of a hypoxia responsive pathway [63,64]. Below this threshold they can relay on suspended animation for survival under anoxia and *hif-1 *is not required [63,65]. However, our findings demonstrate that some globins are upregulated under conditions of total oxygen deprivation. One possible explanation of this finding is that the induction of these genes by hypoxia is quite fast and 'frozen' in this state under anoxia.

Only 6, perhaps 10, out of the 33 globins were anoxia-responsive. Three out of 6 cytoglobin-like and none of the 5 neuroglobin-like proteins were upregulated under conditions of oxygen deprivation, in agreement with most mammalian globin expression studies [39-41,66]. The lack of significant difference in expression under normoxic and anoxic conditions in *hif-1 *mutant worms leads to the conclusion that all anoxia responsive globin genes are regulated by a HIF-1 mediated mechanism. Computational analysis of the globin genomic regions detected the presence of putative hypoxia-responsive sequence elements in all anoxia-induced globin genes, providing additional support for HIF-1-mediated upregulation. These globins are also induced under normoxia in *hif-1 *mutants (Fig. 7), suggesting a more complex regulation that is able to compensate for the loss of *hif-1 *function.

Y75B7AL.1 was mildly induced by anoxia, reaching significance in one set of experiments only (Figs 6 and 7), and required intact *hif-1 *activity for adaptation to anoxia. Prediction programs identify Y75B7AL.1 as a chimeric protein consisting of a G-coupled receptor domain containing 7 transmembrane helices and a globin domain. Its structural properties suggest that this protein may act as an oxygen sensor. The oxygen dependent expression of this globin is surprising since such regulation is unprecedented to date. A chimeric protein comprising a guanylate cyclase and a haem binding domain was recently reported to sense oxygen and to regulate aerotaxis responses [67,68].

### Expression of globin ZK637.13 is regulated by insulin/IGF-1 signaling and not induced by anoxia

Since both dauers and *daf-2 *mutant animals are hypoxia resistant [44] we anticipated that they might constitutively express a subset of globin genes at higher levels. However, we found to our surprise a rather dissimilar pattern of globin regulation in these animals, as summarized in Table 3. Several globins were borderline (not statistically significant) upregulated in dauers (relative to L3), but not in *daf-2 *mutant worms. ZK637.13 was substantially upregulated in *daf-2(e1370) *adults in a *daf-16*-dependent fashion. This globin molecule was expressed at lower levels in L3 and borderline (no statistical support) upregulated in the alternative dauer stage. Interestingly, regulation of transcription of this globin species was not sensitive to anoxia. Since suspended DAF-2 signaling induces enhanced life maintenance it is tempting to anticipate that ZK637.13 contributes to these processes.

**Table 3:** Overview of differential globin regulation. Brackets denote substantial, but not significant, difference in expression

***Globin gene***	**dauer vs L3**	**dauer vs adult**	**L3 vs adult**	***daf-2 *vs N2**	**N2 anoxia vs normoxia**	***daf-2 *anoxia vs normoxia**	***hif-1 *vs N2**	***daf-16 *anoxia vs normoxia**
*C06E4.7*	(up)	up						
*C06H2.5*		(up)	(up)					
*C09H10.8*	(up)	up						
*C18C4.1*	(up)	(up)			up		(up)	(up)
*C18C4.9*				down	up		(up)	up
*C23H5.2*								
*C26C6.7*	up		down	down	up		up	
*C28F5.2*		(up)						
*C29F5.7*								
*C36E8.2*	(up)	(up)	(up)		up		up	up
*C52A11.2*	(up)	up	(up)					
*F19H6.2*								
*F21A3.6*	(up)	(up)		down	up		up	up
*F35B12.8*								
*F46C8.7*		(up)						
*F49E2.4*								
*F52A8.4*	(up)	up						
*F56C4.3*								
*R01E6.6*	(up)	up				up		
*R102.9*			(up)					
*R11H6.3*		(up)				(up)		
*R13A1.8*	(up)	up			up	(up)	(up)	(up)
*R90.5*		up	up					
*T06A1.3*		(up)						
*T22C1.2*	(down)	(down)	down	(down)	up		up	up
*W01C9.5*	(up)	up			up		up	up
*Y15E3A.2*								
*Y17G7B.6*					up		up	up
*Y22D7AR.5*	(up)	(up)						
*Y57G7A.9*		(up)						
*Y58A7A.6*	(up)	(up)						
*Y75B7AL.1*					up		(up)	up
*ZK637.13*	(up)		down	up				

### Multiple pathways control expression of the anoxia responsive globin genes

Surprisingly, none of the HIF-1 responsive globin genes was induced when *daf-2(e1370) *mutant animals were exposed to 12 h of oxygen deprivation (Table 3). Thus induction of these genes by anoxia requires both HIF-1 and DAF-2 function. This could indicate that HIF-1 mediates induction of these genes upon anoxia treatment and that HIF-1 activity is modulated by insulin/IGF-1 signaling, or that HIF-1 and DAF-2 each act to regulate the expression of these globin genes.

The data presented in Figures 10 and 11 provide support for the first model: reduced levels of *hif-1 *mRNA were measured in animals lacking *daf-16 *function and the induction of the globins in response to anoxia was weakened in these mutants. The finding that anoxic treatment fails to induce the HIF-1 responsive globin genes in animals lacking *daf-2 *function leads to an apparent contradiction, however: DAF-16 activity which is negatively regulated by DAF-2 is expected to be fully active in these animals and *hif-1 *would be expected to be fully expressed. Yet the globin genes were not induced upon anoxic treatment in these mutants. To explain this we propose a model in which DAF-16 opposes the expression of these globins, directly or indirectly. Direct inhibition could result from competition of HIF-1 and DAF-16 for the HIF-1 responsive promoters. This explanation would imply that DAF-16 can bind to but not activate these promoters. This is opposite to globin ZK637.13 which is clearly induced by DAF-16 but not HIF-1.

## Conclusion

This work provides the first comprehensive analysis of the globin gene family of *C. elegans*. We illustrate the remarkable structural diversity of this gene family, pointing to a functional variety. In this study we demonstrate significant differential expression of the *C. elegans *globin gene family upon anoxia. Studying expression profiles in different mutant worms has enabled us to provide initial evidence of linked HIF-1 and Ins/IGF-1 signaling for globin regulation under severe hypoxic conditions in the nematode.

We assume that in a natural environment HIF-1 responsive globins are "on" when DAF-2 is "on" i.e. when the conditions for growth and reproduction are favorable. Scarcity of oxygen in otherwise permissive conditions is remedied by the action of HIF-1. Adverse conditions for growth and reproduction (crowding, high temperature,...) lead to reduced DAF-2 signaling and increasing silencing of the HIF responsive globin promoters by the DAF-16 FOXO transcription factor. In contrast, globin ZK637.13 is induced and may contribute to the life maintenance program that is deployed under these conditions.

## Methods

### Culture techniques

The wild-type strain N2 and the mutant strains *daf-2(e1370)*, *daf-16(m26)*, *daf-2(e1370);daf-16(m26) *and *hif-1(ia04) *were obtained from the *Caenorhabditis *Genetics Center. Synchronous populations were initiated from eggs prepared by alkaline hypochlorite treatment of gravid adults and grown at 24°C on cholesterol supplemented Nutrient Agar (OXOID) plates containing a lawn of freshly grown *E. coli *K12 cells [69]. For the study of life cycle specific globin expression experiments worm samples were harvested after 0(unfed L1), 6(L1), 12(L1), 18(L2), 24(L2), 30(L3), 36(L4), 42(L4) and 48(young adult) hours of growth. When the worms reached the fourth juvenile stage FUdR was added at 200 μM final concentration to prevent progeny production. At harvest, worms were washed off the plates, cleaned using Percoll and dense sucrose [70], flash frozen and stored at -75°C until use. *daf*-*2 *(*e1370*) is a temperature-sensitive constitutive dauer former: L2 larvae enter diapause at 24°C but molt to the normal third larval stage at lower temperature. To prevent dauer formation the cultures were incubated at 17°C and shifted to 24°C after the animals had molted to the fourth larval stage. Dauers were grown by spreading 150,000 eggs, 10^10 ^heat killed *E. coli *cells and 1mg haemoglobin (from a 5% stock solution in 0.1N KOH, autoclaved for 10 min) on 10 cm agar (made up with cholesterol supplemented S buffer, pH 7.0) plates. These conditions induce almost 100% dauer formation. Plates containing less than 99% dauers were discarded. *hif-1 *mutant worms were grown at 20°C.

The worms were subjected to anoxia in the respirometer cells of a six channel respirometer from Strathkelvin (Glasgow, Scotland) equipped with Clark electrodes. Young adult (1–2 days after the L4 to adult molt) animals were washed off the plates. Samples containing 20000–40000 worms in 2 ml of S buffer were transferred to the respirometer cells and sealed with rubber stoppers. The worm suspensions were stirred at the appropriate temperature and the oxygen concentration was continuously monitored. Oxygen concentration decreased rapidly below 0.1 μM (= 3.2 μg/L, which is in the order of about 0.001% of the atmospheric concentration) within 15 min and was stable for the next 12 h. Three replicate worm cultures were grown and 3 samples from each culture were subjected to anoxia to account for experimental variation.

### Identification of globin-like genes

Putative globins and globin domains were identified in the genomes of *C. elegans *and *C. briggsae *as described earlier [1,2,24]. Briefly, putative globins were identified using a library of hidden Markov models [71] listed on the SUPERFAMILY website [72]. Sequences that matched the globin motifs with E-values > 10^-5 ^or shorter than 100 aa were discarded and the remaining sequences were checked employing the WormBase Release WS150 [73]. Orthologous sequences were manually aligned following the procedure used earlier in the alignment of over 700 globins [74]. This procedure is designed to fit the putative sequences to the myoglobin fold [75], the pattern of 37 conserved, hydrophobic residues, including 33 intra-helical residues A8, A11, A12, A15, B6, B9, B10, B13, B14, C4, E4, E7, E8, E11, E12, E15, E18, E19, F1, F4, G5, G8, G11, G12, G13, G15, G16, H7, H8, H11, H12, H15, and H19, the three inter-helical residues at CD1, CD4 and FG4 and the invariant His at F8 [74,76]. Although earlier alignments [74] had suggested that globins were characterized by two invariant residues, F8His and CD1Phe, more recent information on globin sequences [1,2] indicate that other hydrophobic residues, such as Tyr/Met/Leu/Ile/Val can occur at the CD1 position as well as Ala/Ser/Thr/Leu at the distal E7 position, in addition to His and Gln.

### RNA isolation and cDNA synthesis

Total RNA was isolated from harvested *C. elegans *wormsusing the RNeasy Midi Kit from Qiagen (Hilden, Germany), according to the manufacturer's instructions. A DNase I (Zymo Research, Orange, California) digestion step was subsequently performed to remove genomic DNA. First strand cDNA was synthesized using an oligo(dT) primer and a Moloney murine leukemia virus reverse transcriptase (Fermentas, Vilnius, Lithuania).

### Expression analysis

cDNA was used as a template to amplify mRNA from each putative globin in a PCR reaction using gene-specific forward and reverse primers. The reverse transcription reaction conditions were 1 μM forward and reverse primer, 200 μM dNTP, 2.5 mM MgCl_2_, and 2.5 units of Taq DNA polymerase (Qiagen, Hilden, Germany) in a volume of 50 μl. The cycling conditions were 95°C for 2 min followed by 40 cycles of 95°C for 1 min, 54°C for 30 sec and 72°C for 30 sec.

The 5' cDNA end of several sequences was identified using a RACE (Invitrogen, Carlsbad, California) experiment. First strand cDNA was synthesized using a gene-specific primer. A polyC tail was added to the 3' end of the cDNA with terminal deoxynucleotide transferase. The 5' end was then amplified using an oligo(dG) adaptor and a gene-specific nested primer in a PCR of 35 cycles of 94°C for 1 min, 54°C for 30 sec and 72°C for 30 sec, followed by a final extension for 5 min at 72°C. The amplified products were sequenced on both strands using BigDye terminator chemistry and an ABI 377 sequencer (Applied Biosystems, Foster City, California).

### Real-time quantitative RT-PCR

Primers (Invitrogen) were designed using Primer3 software [77] and tested for specificity using NCBI BLAST. The sequences are available upon request.

The targets amplified by the primer pairs were evaluated with MFOLD software [78] in order to control for the formation of secondary structures at the site of primer binding. MFOLD analysis was performed using default settings and 50 mM Na^+^, 3 mM Mg^2+ ^and a temperature of 60°C (which is the annealing temperature of the primers).

Quantitative RT-PCR was carried out using a Rotor-Gene 2000 centrifugal real-time cycler (Corbett Research, Mortlake, Australia) using the Platinum SYBR Green qPCR SuperMix-UDG (Invitrogen). Each reaction contained: 12.5 μl of the Platinum SYBR Green qPCR SuperMix-UDG, 100 nM, 200 nM or 500 nM of forward and reverse primers and 5 μl cDNA (1:40 RNA dilution), to a final volume of 25 μl. Amplification was performed in 0.1 ml real-time PCR tubes (Corbett Research) placed in the 72-well rotor of the Rotor-Gene instrument. The cycling conditions were as follows: 50°C for 2 min, initial denaturation at 95°C for 2 min, followed by 45 cycles of 15s at 95°C, 30s at 60°C, and 30s at 72°C (gain set at 8 for SYBR Green). Following the final cycle, melting curve analysis was performed to examine the specificity in each reaction tube (absence of primer dimers and other nonspecific products). The Rotor-Gene software (version 6.0) allows automatic melting curve analysis for all tested samples in a given run. SYBR Green fluorescence of the generated products was continuously monitored throughout the temperature ramp from 60 to 99°C. The temperature rose in 1° increments with a 5-s hold at each degree. A single melt peak for each reaction confirmed the identity of each PCR product. Each assay included a no-template control for every primer pair. All PCR reactions were performed in triplicate. Real-time PCR efficiencies for each reaction were calculated using the formula: *Efficiency *(*E*) = [10^(1/*slope*)^] – 1, from the slope values given by Rotor-Gene software. Amplicons from each reaction mixture were also analyzed by agarose gel electrophoresis.

### Quantification and data analysis

The threshold cycle (*Ct*) values of the Rotor-Gene software version 6.0 (Corbett Research) were exported to Excel (Microsoft) for further analysis. Reaction efficiency estimates were derived from standard curves that were generated using serial dilutions of the corresponding cDNA. These were then used to transform the *Ct *values to relative quantities, which were normalized using the geometric mean of 3 reference genes identified by the geNorm 3.4 software [79]. The geNorm VBA applet for Microsoft Excel determines the most stable reference genes from a set of genes in a given panel of cDNA samples. We evaluated a set of 8 candidate reference genes, comprising *act-1, csq-1, mua-6, tba-1, mlc-3, pat-10, unc-15 *and T22B11.5. The three most stably expressed genes were used to calculate a normalization factor for each of the cDNA samples. Expression levels of all globin genes were determined in at least 3 independent replicate cultures. Differential gene expression was considered significant when the 95% confidence interval of the mean expression levels did not overlap (equivalent to P < 0.05).

### Analysis of potential regulatory elements

All 33 globin genomic regions comprising 2000 bp upstream of the translation initiation sites, all introns and 500 bp downstream were evaluated for the presence of putative HIF-1 (Hypoxia Responsive Elements, HRE, consensus motif RCGTG) and DAF-16 (TTG/ATTTAC and TGATAAG, [80]) binding sites in both directions. Repeats were masked using RepeatMasker.

## Competing interests

The author(s) declares that there are no competing interests.

## Authors' contributions

DH and JVR conceived and designed all experiments; DH, EG and AV performed experiments, DH, EG, LM, SD, SV and JVR analyzed data; DH and JRV wrote the manuscript. All authors read and approved the final manuscript.

## Supplementary Material

Additional file 1 Alignment of the 33 *C. elegans *globins Click here for file

Additional file 2 Globin expression throughout the life-cycle using RT-PCR Click here for file

Additional file 3 Expression levels of globin genes in third stage larvae (L3) and the alternative dauer (diapause) stage, relative to expression in young adult animals. The expression ratios are the average values from 3 replicate cultures (biological repeats). Bars indicate standard error of the mean. Click here for file

Additional file 4 Expression levels of globin genes in young adult wild-type animals following 12 h of anoxia, relative to expression in normoxia. The expression ratios are the average values from 3 replicate cultures (biological repeats). Bars indicate standard error of the mean. Click here for file

Additional file 5 Expression levels of globin genes in *daf-2 *young adults under normoxia or following 12 h exposure to anoxic conditions, relative to wild-type young adult worms under normoxia. The expression ratios are the average values from 3 replicate cultures (biological repeats). Bars indicate standard error of the mean. Click here for file
