# Supplementary material for: Wide diversity in structure and expression profiles among members of the Caenorhabditis elegans globin protein family
Source: BMC Genomics. 2007 Oct 4;8:356. doi: 10.1186/1471-2164-8-356 (PMC2228317; doi:10.1186/1471-2164-8-356)
Supplement: Additional file 1 — Alignment of the 33 C. elegans globins [file 1471-2164-8-356-S1.pdf]

```
              --aaaaaaaAaaAAaaAa--------bbbbbBbbBBbbBBbbcCccccc---------D-------------dddddddeeeEeeEEeeEEeeEeeEEe-----------------FffFffffFF
              ---------|--||--|--------------|--||--||---|--------------|--|--------------------|--||--||--|--||------------------|--|---||
ZK637.13    : -MSMNRQEISDLCVKSLEGRMVGTEAQNIENGNAFYRYFFTNFPDLRVY---------FKGA-------EKYTADDVKKSERFDKQGQRILLACHLLANVY------TNEEVFKGYVRETINRHR
F49E2.4     : EITDEEVTAIRDVWRRAKT---------DNVGKKILQTLIEKRPKFAEY---------FG-I-----QSESLDIRALNQSKEFHLQAHRIQNFLDTAVGSL----GFCPISSVFDMAHRIGQIHF
R102.9      : ELTTDEMQAVRDAWKRAKE---------REIGKHILRALIERKPQ-------------FKDY---FGIHVDEKNHDVYSCREFQLQAHRIQNFLDTAVSSL--------------GFCPIGNIHQ
C52A11.2    : ALNKKDRTLLRETWQRLDD-------PKDIVGLIFLDIVNDIEPDLKKV---------FG-V-------DRAPRAAMLKMPKFGGHILRFYEFMEQLTSML---GTSENLTGAWQLVRKTGRSHV
C28F5.2     : SLTFSQKQALNLSWRLLPQ--------ASACFRKIFLELEIASPKVKQIFYKAALVDAFNK--------------DDDNSATMEVHIKLTTKFFDELLVSL------DDETEFVNKIRGIGSAHA
W01C9.5     : QLTPSQVSVVRRSWRHINT------KGLIIVLTRCFSRLESNCPIVSQC---------FQSA-------TYSLSTNPNGVRTVADHAKYLLQLLDKIIEGD----------VDSEFLREIGANHV
C29F5.7     : PLNAKTKKLVIQEWPRVLA-------QCPELFTEIWHKSATRSTSIKLA---------FGIA-----ENESPMQNAAFLGLSSTIQAFFYKLIITYELNDD----------QVREACEQLGARHV
F19H6.2     : FLTRRERILLEQSWRKTRK------TGADHIGSKIFFMVLTAQPDIKAI---------FG-L-------EKIPTGRLKYDPRFRQHALVYTKTLDFVIRNL------DYPGKLEVYFENLGKRHV
R01E6.6     : PISAQGREIITQCFENPHS----------EFANKVVQRIFEKR-EDYQKYIMN-----L-------------GKERSSIVNNRLKQLVEDIVAHIHDADFI------------ESVSKQYGEEHV
C36E8.2     : ILSVNQRQIIKGCMDNSKD----------DLGERIFRRALERR-DDFKQ---------FI-------------------DNLSKGQRYENSQYLKQFLLGI--VENIMDIDEINRISEEFGCNHV
F46C8.7     : RLSKIQKRAIRFTWHRLQT--RNGGKRVENVFEEVFDKLVKNLPNIRDM---------FST-------RMFLCAMSRGTTSTLRDHSKNCVKMIDSVIKNFDVEKSKRTDTSSENDPRVIGRAHS
R13A1.8     : LIDKESCEVVADSWRLVES-RSSAAETSACFGLFVFQRVFSKIPMLRPL---------FG-L------SESDDVFDLPDNHPVRRHARLFTSILHISVKNV-----DELEAQVAPTVFKYGERHY
C06H2.5     : HLSPHQVQLLTSTWPRIKT--------QSSLFTQVFKVLMQRSPVCREM---------FQKM-------SIVGGFSSNSVCDLNSHTKLLCELLDSLMTDL-----HQPAKIVLAKCQDVGAAHV
C26C6.7     : PLTCAQIHLVRALWRQVYT-----TKGPTVIGASIYHRLCFKN-VMVKEQ--------MKQV--------ELPPKFQNRDNFIKAHCKAVAELIDQVVENL------DHLDNVTGELMRIGRVHA
C18C4.9     : HLTQPQILFVRKTWNHARN------QGALEPAISIFRNSFFKNPEIRQMIM-------FGT--------------KNEGHERLKKHAQLFTVLMDDLIANL------DSPSATVAGLREAGEKHV
F52A8.4     : EPNVYEKELLRRTWSDEFD-------NLYELGSAIYCYIFDHNPNCKQL---------FPFI-------SKYQGDEWKESKEFRSQALKFVQTLAQVVKNI------YHMERTESFLYMVGQKHV
T22C1.2     : ILNSYQKSIVRNAWRHMSQ------KGPSNCGSTITRRMMARKSTIGD----------IL------------------DRSTLDYHNLQIVEFLQKVMQSL------DEPDKISKLCQEIGQKHA
C18C4.1     : RIVDDDFELARTHWIQLQK-----SNKQGLAIRGCFLTMLEKYPQVRPIWG-------FGK---RIEGRGDETWKPEIVEDFYFRHHCASLQAALNMIIQN-----KDDKSGMRRMLNEMGAHHF
R11H6.3     : NLKHEHIRALKTTWARLCE----PPRANCKGIVSLVERVWEKL-DTKDKDVRNI----FYNAAFVDSMHERCERRRSGSIATLRDHTHFFVSLVSQVVSSL-----EKEPAKILEHLDHIGQSHA
C23H5.2     : RFSQEEKDILRRSWKVLDK-------NLNHTAYNIFEMIFNQSPDTRQL---------FPFM----------KFNTGGRSKEIEFHALRFMQVLESVVKTL------DNPETLNPLCDNLGRVHG
F56C4.3     : SFTQEEKNDLEHSWNLVEG-------KKNHIACDIYEMIFNQCPEARRL---------FPKL-------KFVGSKPDRKNNEFAFQAMRFMQVIEGAVKAL------DHLTSLDVILDNLGRRHG
T06A1.3     : IDSYRDFFTLKNWWKSVDR-------KRVEASTYMFSKYLNDFPQNKDL---------YLKL----KNVNAQTVDMNCSDPGFEAIAAQYLKVFDDVITAV--EEKPGDVQTACDRLQAVGKMHR
Y17G7B.6    : NLSVKQKKLLRQSFNAMNS-----GGTFLKLMEKIFRRLETKCPDMRSI---------FLT--TAFVNSLSRERQTPPLVKTEYDHCKCMVGIFERLIENL------ENINEQLTMIRHYGEKHA
Y57G7A.9    : RRPKLDIDRVRSVWMDHIN-------GNDQYFQEVIHRICKRN-EGIRCA--------MLAPNAQHAESVAEEDFVLSNIADRISQFFHQLVEDDVLMDTV----------ELKKACYDLGRQHS
Y15E3A.2    : TISPEHQKLIKRSWNRIPK------AQFGRASLEAFITAAQV-THAI-----------FVD------------------KETENRHVKYFVDLVQSCVDNL-----ENLETGVKPWLDLIGRGHA
Y58A7A.6    : GLSRDDKRIIETCWFKCSQ------KQLRKCSCDMFWDILHTD-EDILRL--------FR-L-------DHVAPNRLKDNDYFKSHASNLALVLNLVVTNL-----QDNFDQAQDALQALGYQHL
R90.5       : GLSPYQQKLLVQCWPNIYT-----TGASGPFANSLYSTLSSRNAKAKELLAKADGVAVFSK----------------SDFDCSVMHCRVTVEILDTVIKNL-----DNDHARITQYLTEIGKQHR
Y75B7AL.1   : QLLGDRLSILKSSWEKANE------MTNGEIGVRVAWNMVRKHPNLCKNDEPEK----VSLL--------NGSCKRSIDHAKFQEIGGRITSFISELLELM---QNNQPESYIVMRIRRVGAVHY
F35B12.8    : ICEQEEVNKMIESYQKIDD--------KYALFEQMFLTIFLEQ-EVEMAYS-------FG-L-------ENLNEQQLKVEQKFRTHVGKFQRFITGIIDML--SKGVESSDQIVEILRIVGRQHG
F21A3.6     : RLSDRQRDVLQKTFAPILQ-------DCVRNGLKIFVRLFSEYPRYKLIWPQ------F----------RAIPDSSLMNAVELRRHASVYLNGLGKIIDSM------RDEEALGKSMSRIAVAHI
C06E4.7     : NLSVAERQCICASWEKAST--------QSDIGCELVARLLNDN-RTRFRALLECKSGSFLG-------SGNYTTEDVNGMKRARSVADGVNCFFNKVISKL---MDANYVEEIQDLSLQLGAMHF
C09H10.8    : HDPQLLSDAISEAWLKSAE-----------LTPTWVWALVDL-PETMHATYKENAQ--FLNI--------------INQVRDFLNILIKVHKCDPEMIKTL-------------SFRLGARHRHY
Y22D7AR.5   : HLTPIDREILNKSWGIVSK-------DMQQVAVNIFQMIFEQAPDAKLM---------FSFM--------MKDYKEDKKSNEFIFHAVRFLQVIESTMTHL------EDPAQLDAVFLNLGKIHA
```

```
            fggg-------G----ggggGggGggGGGgGGggg----------------------------------------hhhhhhHHhhHHhhHhhhHhhhhhhh------
            -----------|--------|--|--|||-||-------------------------------------------------||--||--|---|-------------
ZK637.13  : IYK--------M----DPALWMAFFTVFTGYLESVGC-------------------------------------LNDQQKAAWMALGKEFNAESQTHLKNSNLPHV-
F49E2.4   : YRG--------V--NFGADNWLVFKKVTVDQVTTGTTDSSKEKEDTNSNGTANGKVDTDASLIPIADINNVYSGENCLARLGWNKLMTVIVREMKRGFLEEAMRNC-
R102.9    : MAYRIGQ----I------HFYRGVNFGADNWLTFKKV--------TVEIVTKDCGNSESSSMDLKSVPSLFPSSSSTVIIIGWEKFMSSVIREMKKGFLDEARRNC-
C52A11.2  : RQGFLEQNQNQMEKNYFEIVINVFIERLIPFLTGEQE-------------------LPSSEGKENKKVRFAQNYTTSQITDVWKKFLNTVISQMTDSFELERAKQK-
C28F5.2   : ILAKGSN----F----SSDIWERLGEIAMERVCSHEV-----------------------------------VTKTREASRAWRTLIAILIDELRGGFEGERQHRK-
W01C9.5   : CLKHESG----F----STQEWDRFQEIMVEVILKQDG-----------------------------------VKQSKETSRAWRLLICSFIELIRDGFDAQVRQFR-
C29F5.7   : DF---------ISRGFNSHFWDIFLVCMAEKIDETLS------------------------------AYIPDEDKRNEMILAWQRVINSIVHQMRNGYSDRRKQQL-
F19H6.2   : AMQGRG-----F----EPGYWETFAECMTQAAVEWEA------------------------------------NRQRPTLGAWRNLISCIISFMRRGFDEENGKKK-
R01E6.6   : ELKQYG-----F-KPDFWVAVADAMTLEGVILDMANQ-------------------------------------HPADTVSAWSSLVTMIFSSVRDGYYSELRRHR-
C36E8.2   : QFRANG-----F---KPDFFACTADAVTTECTFLDQA-----------------------------------AHPTSETAAAWSLLTSHVFSAVRDGYYAELRRQR-
F46C8.7   : ILKPYG-----L----AGNYWEKFGEVMIDVVLAQEA-----------------------------------VRDLPGAGQAWVIFTACLVDQMRAGFDENRKTDH-
R13A1.8   : RPD--------ITPHMTEENVRVFCAQIVCTVFDFLR----------------------------------DTEATPKCAESWIELMRYLGQKLLDGFDFAKLTAE-
C06H2.5   : NMNEKCCG---V-------VFDQLGEAFTELITKVEC-----------------------------------VRSKREAVKSWMCVISYMADSIKSGYMEEWAKKR-
C26C6.7   : KVLRGE-----L----TGKLWNTVAETIIDCTLEWGD----------------------------------RRCRSETVRKAWALIVAFVIEKIKAGHHEQRKLML-
C18C4.9   : WPTRNQ-----YGCPFHAHLLDQFATAMIERTLEWGE----------------------------------KKDRTETTQRGWTKIVLFVTEQLKEGFQDEQKRAR-
F52A8.4   : KFADRG-----F----KHEYWDIFQDAMEFALEHRLS-----------------------------IMTDLDDNQKRDAVTVWRTLALYTTVHMRNGFIDGGLKGVN
T22C1.2   : KYRRSKGMK--I------DYWDKLGEAITETIREYQG-------------------------------WKIHRESLRAATVLVSYVVDQLRFGYSRGLHVQGSRDT-
C18C4.1   : FYDACEPH---F------EVFQDSLLESMKLVLNGGD------------------------------------SLDDDIEQSWICTSLRIPPGSFEYDNSK------
R11H6.3   : YLKRYG-----F----KSSHWEKVGEYFVDHVVIQDC-----------------------------------VRGFPEACRAWTVLVSSIVDRLRAAPRRGSFLNS-
C23H5.2   : RLSESRG----F----RTHHWGVFIECTLFHFRKVLG----------------------------QDTYFHRMDALDKVIINWRIIIRLLIKQMKRGFNTDIKNRQ-
F56C4.3   : KLEVNGK----F----RSYYWSVFLECSIYCLRHAFS-------------------------------KRMNDKEVDHVIILWRYLLRDVMKKIKAGTTADIAHRL-
T06A1.3   : QK---------V-SGMDGTMFQNMEEPFIQMVSHILQ-----------------------------------DRFNEKAEMLYRKFFQFCLKYLLEGFNG-------
Y17G7B.6  : QMAESG-----F----TGAMIEQFGEISVFVIGSQDV-----------------------------------VKFNHETVKAWRLLLACVTDEMKVGFDRMSRING-
Y57G7A.9  : SYSKQQ-----F----KMSYWEEFTLTMMGVLEQNYP------------------------------------ETTKEEQKAWLHFLRFVNENMLDGYLDAISRSN-
Y15E3A.2  : NFK--------I----TGKHWEKFGESLLTTATEWNG----------------------------------PGRRHKETVKAWMVMSSFLADRLAHASRLAHHSPM-
Y58A7A.6  : HL---------I----DRTHFQTMYWDIFTDCFE-----------------------------------------RNPPPSFKKGAEREVALKFHRF----------
R90.5     : HLKAEG-----L----SSAVWDDLGDTIMDCARRRCE----------------------------------AVRKHKELRRAWLAIIAYIMDNLKQGQSMTRSSST-
Y75B7AL.1 : DKG--------I--VFTSSVWKEFKHTIQTIISEVQF---------------------------------SSPQEREAALDAWNIFISFIIREMKMGIWAIGDTIG-
F35B12.8  : NVRTMS-----F----TAEKWLIFKNVLLDLLCKDA---------------------------------------NEKVGATWNKLISFMISEVKDSYLEHVRHAR-
F21A3.6   : KWN--------V----QRNHVIHMIEPVLEVVKECNG-----------------------------------YQLDDETRQAWTVLYQVIADLIEVFRCRALND---
C06E4.7   : RMK--------V--WFQAENWLCVKNCLLDSVVSALL-------------------KDTKGTYVICGGLKKVQTVEKHTTHAWFKFVQFIIQNMKKGFLAEALNSD-
C09H10.8  : MNEGNDNC---F----WAPFAQQLPIAMSKMYMRVVT------------------EDSKIRRILRIRSRAAEKSEEEEVCESWRQFSCMLIESMKRGYEGCASEKT-
Y22D7AR.5 : KHEEQ------L--GFSAHYWSVFKECVLFHFRKAMK-----------------------SHNKFHKRNEMSFAEIDSAIILWREVLRFIIDRMKVGYSESGAIRK-
```
