# Supplementary figures and images for: Wide diversity in structure and expression profiles among members of the Caenorhabditis elegans globin protein family
Source: BMC Genomics. 2007 Oct 4;8:356. doi: 10.1186/1471-2164-8-356 (PMC2228317; doi:10.1186/1471-2164-8-356)

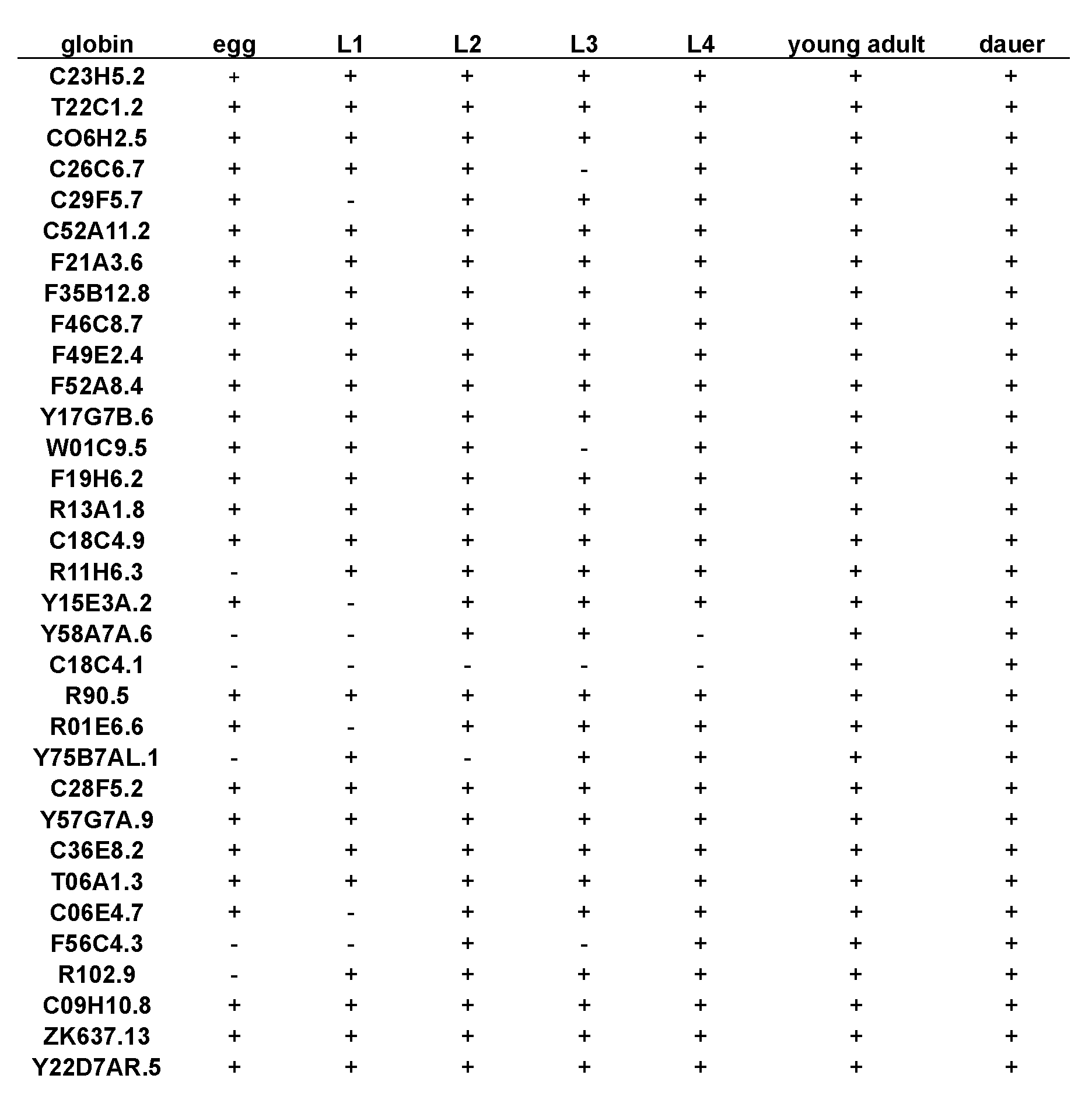

Supplement: Additional file 2 — Globin expression throughout the life-cycle using RT-PCR [file 1471-2164-8-356-S2.tiff]
